# Multimodal mechanical stimulation reduces acute and chronic low back pain: Pilot data from a HEAL phase 1 study

**DOI:** 10.3389/fpain.2023.1114633

**Published:** 2023-04-26

**Authors:** Amy L. Baxter, Anderson Thrasher, Jena L. Etnoyer-Slaski, Lindsey L. Cohen

**Affiliations:** ^1^ Pain Care Labs, Atlanta, GA, United States; ^2^ Department of Emergency Medicine, Augusta University, Augusta, GA, United States; ^3^ Kaizo Clinical Research Institute, Rockville, MD, United States; ^4^ Department of Psychology, Georgia State University, Atlanta, GA, United States

**Keywords:** back pain, chronic pain, opioid, stimulation, thermomechanical stimulation, patient-reported outcome, focal-mechanical vibration

## Abstract

**Background:**

Effective non-opioid pain management is of great clinical importance. The objective of this pilot study was to evaluate the effectiveness of multimodal mechanical stimulation therapy on low back pain.

**Methods:**

11 female and 9 male patients aged 22–74 years (Mean 41.9 years, SD 11.04) receiving physical rehabilitation for acute (12) or chronic (8) low back pain chose heat (9) or ice (11) to accompany a 20-minute session of mechanical stimulation (M-Stim) therapy (Registered with Clinicaltrials.gov NCT04494841.) The M-Stim was delivered in 12 possible repeating “therapy cycle” patterns by three vibration motors (50 Hz, 100 Hz, 200 Hz) with amplitudes between 0.1–0.3 m/s^2^. Ten patients used a contained motor chassis attached to a thermoconductive single-curve metal plate. The next 10 patients' device had motors attached directly to a multidimensionally curved plate.

**Results:**

Mean pain on a 10 cm Visual Analog Scale (VAS) with the first motor/plate configuration went from 4.9 ± 2.3 cm to 2.5 ± 2.1 cm (57% decrease, *p* = 0.0112), while the second reduced pain from 4.8 ± 2.0 cm to 3.2 ± 1.9 cm (45%, *p* = 0.0353). Initial pain was greater with acute injury (5.8 ± 2.0 cm vs. 3.98 ± 1.8, *p* = 0.025) and for patients older than 40 (5.44 vs. 4.52), but pain reduction was proportional for chronic and younger patients. There was no significant difference between plate configurations.

**Conclusions:**

A Phase I clinical pilot investigation on a multi-motor multi-modal device was promising for drug free pain relief. Results suggested pain relief independent of thermal modality, patient age, or pain chronicity. Future research should investigate pain reduction over time for acute and chronic pain.

**Clinical Trial Registration:**

https://ClinicalTrials.gov, identifier: NCT04494841.

## 1. Introduction

Chronic low back pain is a significant contributor to depression, lack of mobility, and decreased quality of life, affecting almost 40% of adults in their lifetimes (1). Opioids are frequently prescribed, particularly with the initial acute injury. As up to 80% of opioid use disorder (OUD) begins with pills prescribed for pain (2), an alternative pain relief option is critically needed. Likewise, for low back pain patients wanting to or needing to wean off opioids, fear of pain is a barrier (3). An effective drug-free solution for both acute and chronic pain could lower prescribed opioids in circulation and assist dependent patients trying to wean.

Heat, ice, acupressure, myofascial trigger point massage, whole body vibration, low frequency focal vibration, and high frequency vibration with and without ice all reduce low back pain (4–7). Recent taskforces evaluating effective chronic pain solutions support a multi-modal biopsychosocial approach; any single treatment, whether pharmacologic, physical, or psychosocial, rarely exceeds a 30% reduction in chronic pain (8, 9). Providing resources for management of pain increases self-efficacy and reduces catastrophizing, two factors associated with lowering opioid use (10–12). Currently, no multi-modal, patient-controlled drug free pain management device exists.

This pilot project sought to evaluate an external mechanical stimulation (M-Stim) gate control-based pain relief device consisting of multiple vibration motors in an array on a thermally conducting metal plate configured to be in direct contact with the low back. In addition to the neuromodulatory M-Stim, patients could choose heat or cold to elicit local and central pain relief *via* central pain modulation (cold) and reduction of spasm and central sensitization (heat). The objective in the current pilot was to demonstrate 50% pain reduction for patients with acute or chronic low back pain after 20 min of use, and to observe patterns to optimize for a full randomized controlled trial of mechanical stimulation vs. electrical stimulation. Ongoing follow-up studies of acute and chronic low back pain control for catastrophizing, Pain Interference, Pain Intensity, and Depression.

## 2. Materials and methods

### 2.1. Participants

In this prospective, single-blinded pilot study patients were recruited at a physical therapy and rehabilitation center in an office complex outside a large inner city metropolitan area. Acute and chronic low back pain patients seeking care were identified by their clinician and offered the opportunity to participate in a study on a new low back pain device prior to initiation of physical rehabilitation interventions for the day. Study personnel verified eligibility and completed informed consent. Inclusion criteria included age 18–90, a self-report of pain greater than 4 on a 0–10 self-report numeric pain rating scale, and the capacity to understand the relevant risks and potential benefits of the study. Exclusion criteria included radicular pain reflecting a potentially mechanical etiology, BMI greater than 30 (for whom the prototype device would not fit), sensitivity to cold or vibration (Raynauds, CRPS, or sickle cell disease); diabetic neuropathy, new neurologic deficits, or skin lesions to the low back area. Patients were informed that they would be giving feedback on design and usability of a pain relief device but they were blinded to the pain reduction hypothesis.

### 2.2. Procedure

After informed consent, study personnel gave enrolled patients a choice of heat, ice pack, or no thermal intervention. A frozen solid ice pack (VibraCool®, Pain Care Labs, Atlanta Georgia) with 4–22 g sections or a microwaved single chamber 150 g clay pack were inserted behind the metal plate, and the device was attached to the patient over a single layer of thin clothing. Patients were instructed how to toggle between 12 different vibration frequency patterns, with five different intensity settings for each pattern. Patients then rated their pain using a 10 cm 0–10 Visual Analog Scale and chose the mechanical stimulation pattern they preferred. Patients then began completing demographic information and medical history while the device was in operation, but they could change the stimulation pattern at any time. After at least 20 min, the device was stopped, and patients recorded a post-treatment pain score. Patients also gave human factors feedback on usability, mechanical stimulation pattern preference, comfort, suggested improvements, whether they would recommend the device, pain relief on a 1–7 Likert scale ranging from “No relief” to “Complete relief”, and design parameters.

**Figure 1:**
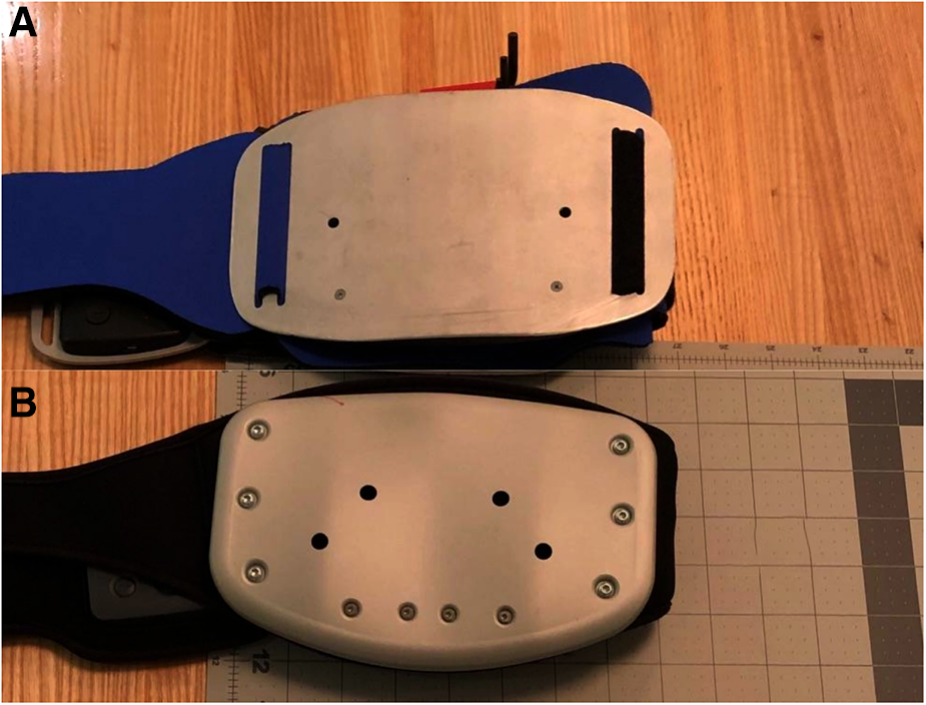
Device design iterations: (**A**) the motor chassis is attached to a thermoconductive single-curve metal plate (**B**) motors are directly attached to a rounded multi-curved plate..

Ten patients used a contained motor chassis attached to a flat thermoconductive single-curve metal plate (Figure 1A). After analysis of the feedback showed a desire for more intensity with mechanical stimulation, the next 10 patients' devices had motors attached directly to an aluminum plate. In response to concerns that the initial plate was too big or that the edges were sharp, the second device had the same dimensions in a multi-curved configuration with a rounded circumferential lip (Figure 1B). The second ten patients were allowed to keep the device for a week and were asked to keep a diary of duration of use and pain relief after one week. Five patients were verbally asked to text when their pain returned to pre-treatment levels.

For the pilot study, no *a priori* power analysis was performed. Statistical analysis was conducted within Excel (Version 2210 Build 16.0 2021 Microsoft Corp). Mean and standard deviations are reported for age (<40 years and ≥40 years), cold/hot treatment preference, sex, device plate configuration, and pain chronicity. Pain scores in cm on VAS were reported with standard deviations and *t*-test *p*-values.

## 3. Results

During two non-consecutive weeks (9/2019, 1/2020) 11 females and 9 males aged 22–74 years (Mean 41.9 years, SD 11.04) receiving physical rehabilitation for acute (12) or chronic (8) low back pain were enrolled (Table 1). Mean pain on a 10 cm Visual Analog Scale (VAS) with the first motor/plate configuration decreased from 4.9 ± 2.3 cm to 2.5 ± 2.1 cm (57% reduction, *p* = .0112), while the second motor/plate configuration reduced pain from 4.8 ± 2.0 cm to 3.2 ± 1.9 cm (45%, *p* = .0353), however this difference between plate configurations was not found to be statistically significant (*p* = 0.1094). Initial pain was greater with acute injury (5.8 ± 2.0 cm vs. 3.98 ± 1.8, *p* = .025) and for patients older than 40 (5.44 vs. 4.52), but pain reduction was proportional for chronic and younger patients. Average pain reduction in both groups was 5.3 on a 7-point Likert scale.

**Table 1:** Patient demographic characteristics.

Patient Characteristics	DuoTherm 1 (*N* = 10)	DuoTherm 2 (*N* = 10)
Gender # (%)		
Female	5 (50%)	6 (60%)
Male	5 (50%)	4 (40%)
Not Reported	0	0
Race # (%)		
Black	8 (80%)	10 (100%)
White	1 (10%)	0
Multiracial	1 (10%)	0
Unspecified	0	0
Ethnicity # (%)		
Hispanic/Latino	2 (20%)	0
Not Hispanic//Latino	7 (70%)	10 (100%)
Unspecified	1 (10%)	0
Pain History # (%)		
Acute <3 m	4 (40%)	7 (70%)
Acute on Chronic	1 (10%)	0
Chronic >3 m	5 (50%)	3 (30%)
Unknown	0	0
Age in Years (%)		
20–29	1 (10%)	0
30–39	4 (40%)	5 (50%)
40–49	3 (30%)	3 (30%)
50–59	0	2 (20%)
60+	1 (10%)	0

**Figure 2:**
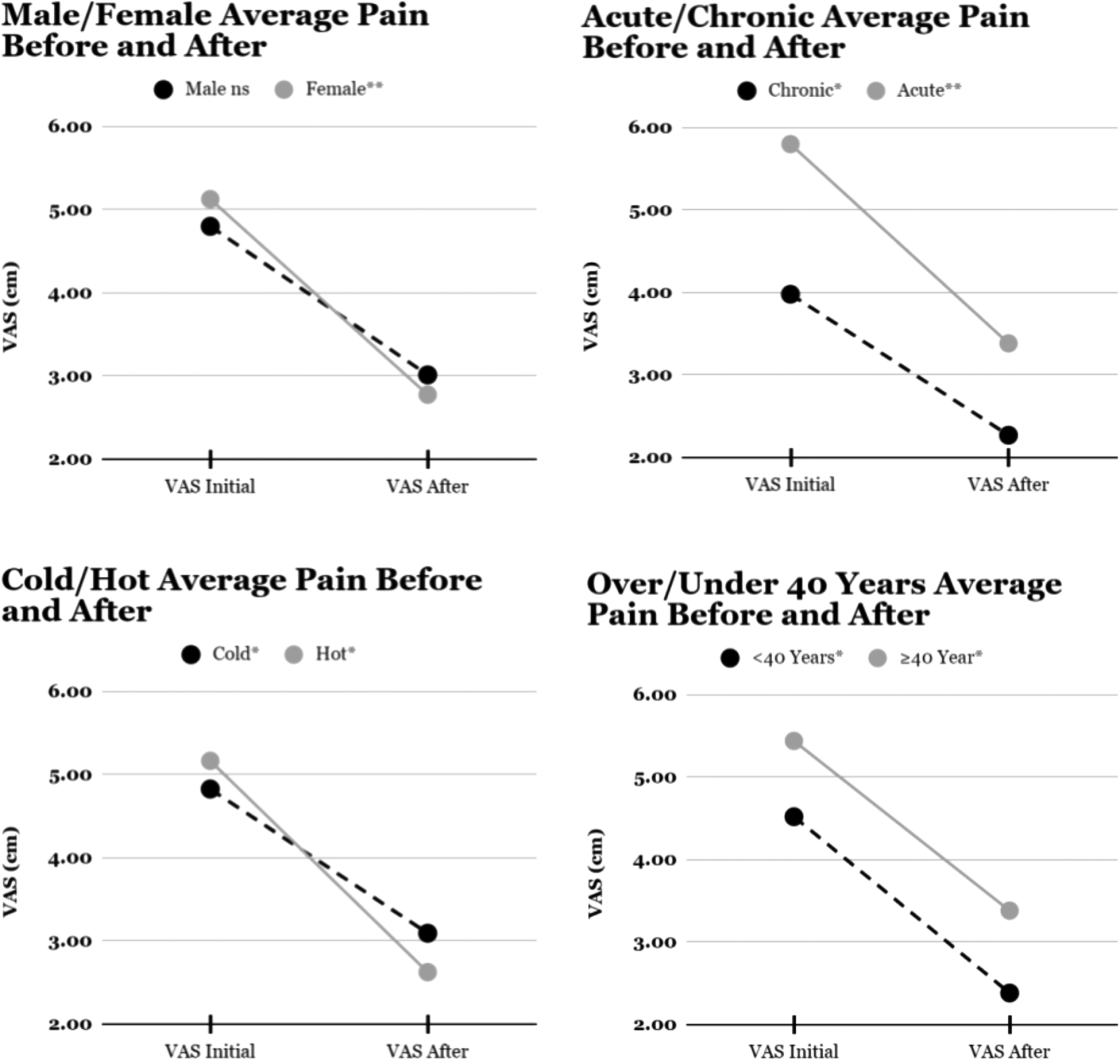
Mean visual analogue scale for pain before and after multimodal mechanical stimulation. *p < 0.05, **p < 0.01, ns *p* > 0.05.

Pain relief was statistically significant overall (*p* < 0.0014), and for all subgroups except males (*p* = .056). Initial reported pain was significantly greater for patients with acute (5.8, SD2.03) than chronic pain (3.98,SD1.79, *p* = .025), but pain reduction was similar amongst all subgroups (Figure 2).

The duration of relief was anecdotally reported (4–4.5 h) by five patients. Eight of ten patients in the second cohort kept a diary of their use of device over a week. Findings included: Pain change on a (1–7) Likert scale continued to improve for all but one who had complete relief after the first session). All eight patients used the device at home, 1 of 4 also used the device at work, 1 of 4 in the car, half used it at home, and half reported use in bed. No preference for vibration therapy pattern was noted and no side effects or adverse events were reported.

## 4. Discussion

This pilot project evaluated an intervention integrating local and central pain interventions including mechanical stimulation, heat, cold, and patient control over intensities and modalities. Initial results demonstrated significant overall pain reduction, with greater initial pain reported by patients with acute pain. Anecdotal and diary results for eight patients suggested that pain relief continued to improve over a week's duration.

The underlying rationale for mechanical stimulation in multiple frequencies is based on new discoveries related to gate control. In 1965, Melzack and Wall hypothesized that stimulation of sensation mechanoreceptors “shut the gate” on pain transmission, an inhibitory mechanism known as “Gate Control” (13). A web of acute pain Aδ nerves transmit nociceptive information to the dorsal column, where the substantia gelatinosa's interneurons prioritize competing sensory information. Fast Aδ, faster Aβ mechanoreceptor and slower C-fiber slow pain signals vie for transmission to the brain for sensory perception. Recent research indicates the principal Aβ-transmitted touch mechanoreceptors respond optimally to different stimulation frequencies: fast adapting light touch Meissner corpuscles detect frequencies between 20 and 40 Hz (14), while fast reacting and long acting deep Pacinian corpuscles begin sensing vibration at 65 Hz, with maximal sensitivity between 180 and 250 Hz (15). With sufficient Aβ stimulation, pain can be overridden, like rubbing a bumped elbow.

In 2017, Hollins et al. reported that Pacinian corpuscles were responsible for 90% of gate control pain relief (16). This finding explains why fast vibratory pain relief was found to be superior to transcutaneous electrical nerve stimulation (TENS) units for musculoskeletal and chronic pain in the early 80's, but failed to be effective when tried with lower frequency interventions (7). By transcutaneous electrical stimulation slower frequencies (typically in the 20–40 range), TENS units stimulate the Meissner corpuscles. While higher frequency TENS (60–120 Hz) may engage the lower range of Pacinian corpuscle inhibition (17), the superficial transcutaneous transmission or insulating adipose tissue may inhibit reaching the deep Pacinian nerves. Multiple metaanalyses on 200 Hz devices since 2009 demonstrate even the sharp pain of needles can be blocked with mechanical stimulation placed proximal to pain (18, 19). In addition to these peripheral effects, recent fMRI research suggests complex cortical and inhibitive nociceptive pain relief from cognitive interventions (20–23).

In addition to activating gate control theory-based pain relief, studies suggest vibration may also benefit low back pain through musculoskeletal mechanisms over time. By improving proprioception and improving muscle strength and size through high frequency vibration (24), pain from sitting and chronic over-stretched lumbar musculature may be reduced. Vibration acts as a mechanical signal that increases cellular anabolic activity, decreasing osteoclast activity, changing gene expression of growth factors, and increasing growth hormone (25). Improved repair could contribute to pain reduction.In 1983, Lundeberg evaluated multiple low amplitude vibratory delivery locations, times, frequencies, delivery areas and pressures for 135 acute and 596 chronic musculoskeletal pain patients (7). For chronic low back pain, 48 out of 60 patients received relief following 30–45 min of peripheral stimulation. Low pressure 200 Hz or moderate pressure 50–150 Hz was most effective, with 80% finding greater relief with unquantified “moderate” pressure. A 10 × 20 cm vibrating foam plate was superior to a small focal delivery area, and the most effective pain relief was directly on the site of pain. In 24 patients for whom TENS had been ineffective, vibratory therapy was effective for 20, with long term and long lasting pain relief for 13, and four discontinuing due to resolution of pain (26). Of note, Lundeberg reported prolonging pain relief by applying ice, and showed that naloxone did not reverse the pain relieving effects, supporting a spinally mediated mechanism (27). Beyond Lundeberg, published research on focal vibration for low back pain is limited to a 2018 study by Lurie et al. involving 18 adult volunteers who stood for 2.5 h. Application of a 53 Hz 22 cm × 18 cm × 3.5 cm thick vibration device relieved pain for 4 times the application time (28).

In our study, we adapted the size of Lundeberg's plate, but used the frequencies found effective for gate control. While frequency is often reported in focal mechanical stimulation research, amplitude depends on how the motors are mounted, measured, and attached to the body, and is rarely reported. The change between our first and last configuration changed two parameters that may have affected amplitude differently. While mounting the motors directly to the plate should have increased the transmission of force, the curvature on the edges of the second plate configuration likely reduces the free movement of the plate, dampening the amplitude. In bench testing, removing the rim, but not the ergonomic configuration of the plate, improved transmission of the vibration to the skin.

## 5. Conclusions

Preliminary data from this pilot of a multimodal thermomechanical low back device showed significant pain relief that persisted or improved with continued use. Interesting findings for future investigations included approximately four-hour reported pain relief in the five patients asked to report duration, and similar pain reduction across groups despite idiosyncratic use of frequency patterns and thermal choices. Phase 2 studies will incorporate an active control, and will use a primary outcome measure of opioid use or initiation in patients who have not had spine surgery, with pain as a secondary outcome. Additionally Phase 2 studies will include measures of catastrophizing, depression, and pain interference with three month follow up for acute use, and six month follow-up for chronic patients.

Our pilot findings suggest a that patient-controlled device that incorporates vibration, heat, and cold might potentially provide a drug-free pain management solution for low back pain. We observed that while acute pain was greater than chronic pain, both responded consistently to the intervention. Given the prevalence and severity of pain combined with our opioid epidemic, these preliminary findings are promising and support future randomized controlled trials.

## Data Availability

The datasets presented in this study can be found in online repositories. The names of the repository/repositories and accession number(s) can be found below: Clinicaltrials.gov ID NCT04494841, https://clinicaltrials.gov/ct2/show/results/NCT04494841?term=NCT04494841&draw=2&rank=1.
